# Regulatory T Cells in *Mycobacterium tuberculosis* Infection

**DOI:** 10.3389/fimmu.2019.02139

**Published:** 2019-09-11

**Authors:** Paula Cardona, Pere-Joan Cardona

**Affiliations:** Unitat de Tuberculosi Experimental, Fundació Institut Germans Trias i Pujol, CIBER Enfermedades Respiratorias, Universitat Autònoma de Barcelona, Badalona, Spain

**Keywords:** tuberculosis, Treg cells, Th17 cells, tolerance, inflammation

## Abstract

Anti-inflammatory regulatory T cells have lately attracted attention as part of the immune response to *Mycobacterium tuberculosis* infection, where they counterbalance the protective but pro-inflammatory immune response mediated by Th17 cells and especially by the better-known Th1 cells. In chronic infectious diseases there is a delicate balance between pro- and anti-inflammatory responses. While Th1 and Th17 are needed in order to control infection by *Mycobacterium tuberculosis*, the inflammatory onset can ultimately become detrimental for the host. In this review, we assess current information on the controversy over whether counterbalancing regulatory T cells are promoting pathogen growth or protecting the host.

## Introduction

Tuberculosis (TB) is a global disease caused by the bacillus *Mycobacterium tuberculosis* (Mtb). According to the World Health Organization, in 2017, 10 million people developed the disease and 1.6 million people died because of it (1). Compounding the situation, approximately a quarter of the world population is currently infected with Mtb (latent TB infection: LTBI) (2).

While progression from Mtb infection to active TB is associated with some known risk factors, including HIV infection and malnutrition, it is still unclear why some apparently healthy people develop the disease. One hypothesis is that poor control of the inflammatory response is the culprit. While an effective immune response is essential to control the infection at an early stage, excessive inflammation may be detrimental later on. The damage-response framework of microbial pathogenesis (3) suggests that both ends of the scale would lead to the development of the disease, where a weak response would benefit the dissemination of the bacilli and a very strong response would favor the lung tissue damage characteristic of active TB. The fact that the majority of cases of Mtb infection develop as LTBI imply that most individuals have intermediate response levels (4). There are diverse studies pointing to the immune balance and the development of TB. The modulation of the leukotriene A4 hydrolase locus, which is key in the balance between pro- and anti-inflammatory eicosanoids, showed to be important in the regulation of TNF-α levels, and thus in the susceptibility to Mtb (5, 6). Interestingly, these authors showed that among individuals with meningitis TB, both homozygous forms of a specific genotype affecting this locus were related to a decreased survival of patients, supporting the idea that both a low and high inflammatory response may be detrimental.

This double-edged nature of the immune response also comes into play in the concept of disease tolerance, a host defense strategy in which less damage is done by the pathogen or the immune response it triggers, although the burden of the microorganism itself is not reduced. In this context, immunosuppressive mechanisms are seen as one of the ways in which the host achieves tolerance (7). Regulatory T cells (Tregs), which suppress, and thus counterbalance the inflammatory response, are one such mechanism.

There is a recurring debate as to whether Tregs are beneficial or detrimental in Mtb infection. Many studies, mainly of blood samples, show higher numbers of Tregs in TB patients than in LTBI subjects or healthy controls. Some of these studies also focus on the follow-up of TB patients undergoing treatment. However, it is not yet clear whether high levels of Tregs are a consequence of inflammation or a risk factor for development of TB. After briefly introducing Tregs, the present review will address this question by examining the available data from animal models and human subjects.

## Regulatory T Cell Characterization

The immune system has mechanisms for suppressing the response to persistent self- or non-self-antigens. Tregs are a lymphocyte subset whose main role is maintaining immune homeostasis and peripheral tolerance.

The key cytokines involved in the immunosuppressive function of Tregs are IL-10, TGF-β, and IL-35 (8–10). By down-modulating the co-stimulatory molecules CD80 or CD86 in a CTLA-4-dependent mechanism, Tregs interfere with T-cell activation by dendritic cells (11). Tregs also express granzymes and thus induce apoptosis of the target cells (12). Another mechanism of suppression is metabolic disruption, achieved by consuming available IL-2 (13).

Tregs might be induced in the thymus during development (tTregs) or in peripheral tissue such as mucosa (pTregs). Research on therapies that modulate these cells, or the administration of *in vitro* induced Tregs (iTregs), have led to efforts to differentiate them (14). tTregs generally target auto-antigens, are more stable, and have higher TCR affinity. The expression of the transcription factor Helios has been linked to tTregs (15) since it is detected in all the CD4^+^FoxP3^+^ thymocytes, but only in around 70% of CD4^+^FoxP3^+^ from peripheral lymphoid tissues. The high expression of the surface marker Neuropilin 1, which is up-regulated by TGF-β, has also been considered as a tTregs marker (16). Interestingly, it had been previously shown that the induction of pTregs, but not tTregs, is dependent on this cytokine (17). Neither of these markers clearly identifies the thymic induced Tregs from pTregs or iTregs.

### Treg Phenotype Markers

Identification of an optimal cellular marker for characterizing Tregs in general is also proving difficult. Some markers used, in combination with CD3 and CD4, include CD25, FoxP3, CD127, or CD39. Although CD8^+^ Tregs have also been described, as recently reviewed by Yu et al. (18), they have not been considered in the present review.

CD25 is highly expressed in Tregs and is in fact related to one of the suppression mechanisms of these cells. However, this extracellular marker, which is the α-chain of the high affinity receptor for IL-2, is also upregulated in activated T cells (19). Therefore, it is generally used in combination with other markers.

FoxP3 is a transcription factor, Forkhead box P3, long used as a marker for Tregs, since it is essential for their development (20). IPEX (immune dysregulation, polyendocrinopathy, enteropathy, X-linked) syndrome is an autoimmune disease caused by mutations in FoxP3 that lead to Treg dysfunction (21). This mutation has also been described in scurfy mice, which have high lymphocytic infiltration resulting in death after a few weeks (22). Nevertheless, FoxP3 has also been associated with other cell types (23), and T cells with regulatory properties that do not express it have also been described (24), so the case for using it as a marker is considerably weakened. Besides, working with an intracellular marker is less appealing and often impractical, since it interferes with functional assays.

In the case of CD127, the α chain of the IL-7 receptor, low to undetectable expression levels have also been used to identify Tregs among CD4^+^CD25^+^ cells (25). This marker is down-regulated in T cells after activation and re-expressed in all but FoxP3^+^ cells. FoxP3 interacts with the promoter CD127 and represses it (26).

CD39 is an ectonucleotidase that turns ATP or ADP into AMP and is expressed by CD4^+^CD25^+^ and CD4^+^FoxP3^+^ cells. This has a dual anti-inflammatory effect: it eliminates the inflammatory mediator ATP and increases production of adenosine, which inhibits the NK- and T-cell response (27). Characterization of CD4^+^CD25^+^CD127^−^ Tregs from peripheral blood mononuclear cells (PBMCs) according to CD39 expression showed that CD39^hi^ Tregs secrete more IL-10 and have a stronger suppressive activity. Moreover, in the presence of IL-1β and IL-6, CD39^low^ Tregs differentiated into Th1 or Th17 and down-regulated the expression of FoxP3 as compared to CD39^hi^ Tregs (28). Further supporting the use of CD39 to characterize a regulatory T-cell phenotype, it has been described that CD4^+^CD25^hi^CD39^+^ cells produce lower levels of IFN-γ and higher of TGF-β than CD4^+^CD25^hi^CD39^−^ cells or CD4^+^ T cells with low or no expression of CD25 (29).

### Th17-Counterbalancing Role

Th17 cells are characterized mainly by the production of IL-17, which promotes the synthesis of other pro-inflammatory cytokines, and chemokines (30). The transcription factor required for the development of Th17 cells is RORγt (31).

Tregs and Th17 cells are closely intertwined. TGF-β induces both FoxP3 and RORγt expression; however, in order to induce Th17, TGF-β also requires the presence of IL-6, in which case the balance is tipped toward Th17 cells instead of Tregs (32). Otherwise, FoxP3 inhibits the function of RORγt (33). It has been shown that CD4^+^CD25^hi^CD39^+^ Tregs have a role in constraining pathogenic Th17 cells (34).

## Tregs and Disease

Tregs have been linked to a number of inflammatory diseases, mostly autoimmune but some of them infectious. Equilibrium with Th17 cells is widely thought to be the key factor in these conditions, but this review will focus on Tregs.

Multiple sclerosis, a chronic autoimmune inflammatory disease of the central nervous system, has been related to an increase in the number and activity of pro-inflammatory Th17 cells and a corresponding decrease in Tregs (35). Rheumatoid arthritis, also of autoimmune origin, affects synovial tissue, where an imbalance has been reported between Tregs and Th17 cells (36).

The relevance of the balance between these cell populations was shown in a Treg phenotyping study carried out in PBMCs in the context of organ transplants. Renal allograft rejection was associated with a high ratio of CD4^+^CD25^−^CD39^+^ cells, which have a pro-inflammatory memory profile, to CD4^+^CD25^+^CD39^+^ Tregs (37).

High levels of Tregs have been associated with different types of cancer, both in peripheral blood samples, and in solid tumors. However, their precise role in cancer is still undefined, with some researchers linking higher levels to a favorable outcome, and others to a poorer prognosis. In addition to these differences in number, higher Treg suppressive activity has also been described in cancer cases (38).

Another area of study is the relationship between Tregs and different pathogens. In *Helicobacter pylori* infection, increased Treg frequency appears to help limit damage due to inflammatory onset, but it also promotes bacterial growth (39). This pattern is quite common and applies to a number of different pathogens, including viruses (40, 41).

The two-edged action of Tregs also plays a role in Mtb infection. The pro-inflammatory response characterized by the production of IFN-γ or IL-17 is necessary to control Mtb growth, so Tregs would appear to be detrimental in terms of countering the pathogen. However, if left unchecked, the inflammatory response ultimately causes excessive tissue damage to the host. Tables 1, 2 summarize TB-related research with a focus on Tregs. These studies of animal models and human cohorts are discussed below separately.

**Table 1:** Summary of data on Tregs and TB from animal models.

**Animal model**	**Sample**	**Treg phenotype**	**Relationship between Tregs and TB**	**References**
Guinea pig, aerosol infection with Mtb H37Rv and Erdman laboratory strains, and 3 clinical isolates of Mtb	Lungs	FoxP3 mRNA	Progression of infection increases Treg levels	(42)
Guinea pig, aerosol infection with clinical isolates of Mtb Beijing strains	Lungs	FoxP3 mRNA	Progression of infection increases Treg levels	(43)
Cynomolgus Macaque, bronchoscopic instillation infection with Mtb Erdman strain	Lungs and LNs	CD3^+^CD4^+^FoxP3^+^	Higher levels of Tregs in Mtb-positive lungs and draining LNs	(44)
	PBMCs		Higher levels of Tregs found prior to infection in animals that did not develop the disease	
Cynomolgus Macaque, bronchoscopic instillation infection with Mtb Erdman strain	Blood	CD4^+^CD25^+^FoxP3^+^	Higher levels of Tregs correlates with less severe lung pathology after IL-2 treatment	(45)
	BAL			
DBA/2 mice, aerosol infection with Mtb Kurono or Erdman strains	Systemic	CD25^+^	Treg depletion decreases spleen and lung BL and improves lung pathology (at early stages of infection)	(46)
C57BL/6 & bone marrow chimeric mice, aerosol infection with Mtb H37Rv strain	Lungs and LNs	CD4^+^FoxP3^+^	Progression of infection increases Treg levels (WT)	(47)
	Systemic	FoxP3^+^	Treg depletion decreases lung BL	
C57BL/6, aerosol infection with Mtb Erdman strain	Systemic	CD25^+^	Treg depletion does not affect BL or lung pathology	(48)
C57BL/6, intranasal infection with *M. bovis* BCG Pasteur strain 1173P2				
C3HeB/FeJ & C3HeN, intravenous infection with Mtb H37Rv Pasteur strain	Spleen	CD4^+^CD25^+^FoxP3^+^	Higher levels of Tregs in strain less susceptible to infection (C3HeN)	(49)
	Systemic	CD25^+^	Treg depletion exacerbates lung pathology (C3HeN)	
I/StSnEgYCit and C57BL/6JCit mice, aerosol infection with Mtb H37Rv Pasteur strain	Mediastinal LNs	CD4^+^CD25^+^FoxP3^+^	Higher levels of Tregs found in strain less susceptible to infection (C57BL/6JCit)	(50)
			Progression of infection increases Treg levels (C57BL/6JCit)	
C57BL/6 & P25 TCR transgenic mice, aerosol infection with Mtb H37Rv strain	Systemic	CD4^+^CD25^+^	Mtb-specific Treg adoptive transfer increases BL	(51)
C57BL/6, FoxP3-GFP reporter & TCR KO mice, aerosol infection with Mtb H37Rv strain	Pulmonary LN	CD4^+^FoxP3^+^	Early expansion of Mtb-specific thymically derived Tregs	(52)
C57BL/6 & IL-12p35 or IL12p40 KO mice, aerosol infection with Mtb H37Rv strain			Progression of infection decreases Mtb-specific Tregs in an IL-12 dependent manner	
C57BL/6 & TLR2 KO mice, aerosol infection with Mtb Erdman strain	Lungs	CD4^+^FoxP3^+^	Progression of infection decreases Treg levels	(53)
			KO have fewer Tregs than WT after infection, and higher BL	
C57BL/6, BALB/c, DBA/2 mice, aerosol infection with clinical isolate of Mtb Harlingen strain	Lungs	CD4^+^FoxP3^+^	Higher levels of Tregs found in strains less susceptible to infection (C57BL/6 and BALB/c)	(54)
C57BL/6, aerosol infection with Mtb H37Rv strain	Systemic	CD4^+^CD25^+^FoxP3^+^	Treg depletion does not interfere with BCG effect	(55)
C57BL/6, aerosol infection with Mtb Erdman strain or *M. bovis* Wag201 strain	Systemic	CD25^+^	Treg depletion has no effect on BCG-mediated reduction of BL and improves lung pathology	(56)
BALB/c, intratracheal infection with Mtb H37Rv strain	Lungs	CD4^+^FoxP3^+^	Lower number of Tregs correlate with better vaccine protection	(57)
	Systemic	CD25^+^	Treg depletion does not interfere with vaccine effect	

*BAL, bronchoalveolar lavage; BL, bacillary load; KO, knock-out; LN, lymph node; PBMCs, peripheral blood mononuclear cells; WT, wild-type*.

**Table 2:** Summary of data on Tregs and TB from human cohorts.

**Population of study**	**Sample**	**Treg phenotype**	**Relationship between Tregs and TB**	**References**
PTB (*n* = 13) vs. HC (*n* = 12)	PBMCs stimulated with Mtb-RD1 proteins for 1 or 6 d	CD4^+^CD25^hi^FoxP3^+^ or CD4^+^CD25^hi^CD39^+^	Higher levels of Tregs in PTB	(29)
HC (*n* = 9) vs. LTBI (*n* = 8) vs. PTB (*n* = 13)	Cryopreserved PBMCs	CD4^+^CD25^+^ or CD4^+^CD25^hi^	LTBI have higher Tregs than HC, but lower than PTB	(58)
HC (*n* = 28) vs. LTBI (*n* = 20) vs. TB (*n* = 20)	Cryopreserved PBMCs	CD4^+^CD25^+^CD127^−^	Lower levels of Tregs in HC, no differences between LTBI and PTB	(59)
LTBI pre and post ATT (*n* = 20)		CD4^+^CD25^+^FoxP3^+^	Increase in Tregs after prophylactic treatment	
LTBI (*n* = 18) vs. PTB (*n* = 33) vs. cured TB (*n* = 20)	PBMCs	CD4^+^CD25^+^FoxP3^+^	Higher levels of Tregs in PTB, no differences between LTBI and cured TB	(60)
Follicular hyperplasia (*n* = 6) vs. LNTB (*n* = 20)	LN tissue	FoxP3^+^	Expression of FoxP3 in LNTB	
HC (*n* = 23) vs. TB (*n* = 27)	PBMCs	CD4^+^CD25^hi^	Higher levels of Tregs in TB	(61)
		FoxP3 mRNA		
Healthy TB case contacts (*n* = 134) vs. LTBI (*n* = 151) vs. TB (*n* = 126)	Total RNA from whole blood	FoxP3 mRNA	LTBI have higher Tregs than HC, but lower than active TB	(62)
PTB (*n* = 19 in PBMCs and *n* = 6 in BAL) vs. LTBI (*n* = 10 in PBMCs and *n* = 6 in BAL)	PBMCs and BAL	CD4^+^CD25^+^FoxP3^+^	Higher levels of Tregs in PTB (both in PBMCs and BAL)	(63)
			Higher levels of Tregs in BAL than in PBMCs in PTB	
LNTB (*n* = 18)	Cryopreserved LNMCs and PBMCs	CD4^+^CD25^+^FoxP3^+^CD127^−^	Higher levels of Tregs in LNMCs than in PBMCs	(64)
HC (*n* = 66) vs. LTBI (*n* = 27) vs. TB (*n* = 62)	Whole blood	CD3^+^CD4^+^FoxP3^+^	Higher levels of Tregs in TB than HC or LTBI, no differences between these two groups	(65)
PTB (*n* = 35) vs. STB (*n* = 13)			Higher levels of Tregs in PTB	
Pleural TB (*n* = 14)	Whole blood and PFMCs		Higher levels of Tregs in PFMCs	
LTBI (*n* = 20) vs. PTB (*n* = 20) vs. pleural TB (*n* = 15)	PBMCs	CD4^+^CD25^+^FoxP3^+^	Higher levels of Tregs in PTB or pleural TB	(66)
Pleural TB (*n* = 15)	PBMCs and PFMCs		Higher levels of Tregs in PFMCs	
TB (*n* = 31) vs. LTBI (*n* = 30)	Cryopreserved PBMCs stimulated with PPD, ESAT-6 or CFP-10 for 24 h	CD4^+^CD134^+^CD25^+^CD39^+^	Higher levels of Tregs in TB	(67)
PTB (*n* = 48) and/or EPTB (*n* = 28) (before and after ATT) vs. HC (*n* = 83)	Whole blood stimulated with BCG and cryopreserved afterwards	CD3^+^CD4^+^CD25^+^CD39^+^ FoxP3^+^	Higher levels of Tregs in TB, but no differences between PTB and EPTB	(68)
			Increase in Tregs after ATT in PTB	
PTB during ATT (*n* = 20, AFB+ vs. AFB-)	PBMCs	CD4^+^CD25^+^CD127^−^	Tregs increase with TB treatment	(69)
LTBI (*n* = 19) vs. PTB (*n* = 21)	PBMCs	CD4^+^FoxP3^+^, CD4^+^CD25^+^ or CD4^+^CD25^+^FoxP3^+^	Higher levels of Tregs in PTB	(70)
PTB (*n* = 21)		CD4^+^CD25^+^FoxP3^+^	Higher levels of Tregs with higher BL in sputum	
DS-PTB (*n* = 16) and MDR-PTB (*n* = 4) during ATT			Decrease of Tregs with ATT only if DS-PTB	
PTB (*n* = 41) vs. HC (*n* = 24)	PBMCs	CD4^+^CD25^+^FoxP3^+^	Higher levels of Tregs in PTB	(71)
PTB during ATT (*n* = 20)			Tregs increase transiently with ATT and decrease to HC levels	
HC (*n* = 15) vs. PTB (*n* = 10)	PBMCs stimulated with rESAT6-CFP10 during 6 days	CD3^+^CD4^+^CD25^+^FoxP3^+^	Higher levels of Tregs in PTB	(72)
PTB during ATT (*n* = 10)			Treg decrease with ATT	
PTB (*n* = 15) vs. HC (*n* = 17, 41% QTF+)	PBMCs cultured overnight	CD4^+^CD25^+^CD39^+^ or CD4^+^CD25^+^FoxP3^+^	Higher levels of Tregs in PTB, no differences between QTF+ and QTF-	(73)
PTB during ATT (*n* = 12)			Treg levels decrease with ATT	
HC (*n* = 15) vs. PTB (*n* = 13) vs. completed ATT (*n* = 21)	PBMCs cultured overnight	CD4^+^CD25^+^CD39^+^ or CD4^+^CD25^+^FoxP3^+^	Higher levels of Tregs in PTB than HC and cured TB. Treatment failure: increase in Tregs	(74)
LTBI (*n* = 10) vs. PTB (*n* = 10) vs. cured TB (*n* = 10)	PBMCs stimulated with *M. bovis* antigens for 48 h	CD4^+^CD25^hi^FoxP3^+^	Lower levels of Tregs in LTBI	(75)
HC (*n* = 80) vs. LTBI (*n* = 80) vs. PTB (*n* = 90)	PBMCs	CD4^+^CD25^+^C127^−^	LTBI have higher Tregs than HC, but lower than PTB	(76)
PTB (*n* = 30) vs. cured TB (*n* = 10) or HC (*n* = 30)	Whole blood	CD4^+^CD25^+^ or CD4^+^CD25^+^FoxP3^+^	Higher levels of Tregs in PTB	(77)
AFB + (*n* = 21) vs. AFB – (*n* = 9)			Higher levels of Tregs in AFB +	
Cavitary TB (*n* = 19) vs. non-cavitary TB (*n* = 11)			Higher levels of Tregs in cavitary PTB	
HC (*n* = 14) vs. DS-PTB (*n* = 33) vs. MDR-PTB (*n* = 7) vs. stable MDR-PTB (*n* = 16)	PBMCs	CD4^+^FoxP3^+^	Higher levels of Tregs in active PTB (DS or MDR), no differences between stable MDR-PTB and HC	(78)
HC/LTBI (*n* = 20) vs. DS-PTB (*n* = 20) vs. MDR-PTB (*n* = 18)	PBMCs	CD4^+^CD25^+^FoxP3^+^	Higher levels of Tregs in DS-PTB than HC/LTBI, but lower than MDR-PTB	(79)
HC (*n* = 30) vs. DS-TB (*n* = 30) vs. MDR-TB (*n* = 30)	PBMCs	CD4^+^CD25^+^	Higher levels of Tregs in DS-TB than HC, but lower than MDR-TB	(80)

*AFB, Acid-fast bacilli; ATT, antituberculous treatment; BAL, bronchoalveolar lavage; EPTB, extrapulmonary tuberculosis; HC, healthy control; LNTB, lymph node tuberculosis; LTBI, latent tuberculosis infection; MDR-TB, multidrug-resistant tuberculosis; PBMCs, peripheral blood mononuclear cells; PFMCs, pleural fluid mononuclear cells; PTB, pulmonary tuberculosis; QTF, quantiferon; STB, severe tuberculosis (PTB with meningitis)*.

### Tregs in TB Animal Models

In the guinea pig model, while studying the pathogenicity of different Beijing sublineages of Mtb, it was shown that highly virulent strains have increasing levels of FoxP3, IL-10, and TGF-β mRNA in lung tissue as infection progresses (42). In another study with a similar focus, FoxP3, and TGF-β expression was also found to increase in the lungs with progression of infection, but pro-inflammatory IL-17 increased as well. With less virulent strains, the increase is not as steep (43).

Results from a non-human primate (NHP) Mtb infection model suggest that augmented Treg levels are a response to inflammation, rather than the cause of TB disease (44). These authors found that NHPs that failed to develop TB disease (i.e., LTBI cases) had higher levels of Tregs in peripheral blood prior to infection than those that developed active TB. Although all subjects experienced an initial decrease in Treg frequencies, animals that remained latently infected remained at higher levels during the period of decline and eventually returned to pre-infection levels as infection progressed, while Tregs continued to increase in the NHPs with TB. In a separate study, IL-2 treatment of NHP in early-stage infection expanded Foxp3^+^ Tregs, and CD4^+^/CD8^+^/γδ T effector cells (45). The authors linked ensuing resistance to severe TB lesions to an optimal cooperation between IL-2-expanded CD4^+^ T cells and Tregs, by preventing overreacting inflammatory responses.

In a murine model, when CD25^+^ cells were depleted in DBA/2 mice prior to Mtb aerosol infection, infection outcomes improved: there was a reduction in the bacillary load (BL) of lungs and spleen, together with less severe histopathology in the lungs (46). Nevertheless, this effect was seen only at week 2 post-infection, and afterwards there was no difference between groups. There was also no significant effect if CD25^+^ cell depletion was done at the chronic stage of infection. Similar results were observed using a mixed bone chimera system where FoxP3^+^ cells were depleted with anti-Thy1.1 antibodies: there was a reduction in BL in the lungs, but not the spleen, at day 23 post-infection (47).

However, depletion of CD25^+^ cells 3 days prior to infection in both *M. bovis* BCG- and Mtb-infected C57BL/6 mice had no effect on BL, even though a reduction in CD4^+^CD25^+^ cells was observed for at least 23 days (48). In fact, the characterization of two mouse strains with the same haplotype but different susceptibility to Mtb infection (C3HeB/FeJ and C3H/HeN) showed that the more susceptible animals had lower numbers of Tregs (49). Furthermore, after CD25^+^ cells were depleted in the more resistant strain, lung pathology became more severe. Another study in two mouse strains with different susceptibility, the hyper-susceptible I/StSnEgYCit and the relatively resistant C57BL/6JCit, showed that Treg levels were lower in the mediastinal lymph nodes of the susceptible mice and remained practically constant during the follow-up period (until week 17 post-infection), while this cell population increased over time in the resistant C57 mice. The authors concluded that this is a host protective strategy (50).

Scott-Browne et al. (47) failed to see a similar percentage increase, but did find an increase in total numbers of CD4^+^FoxP3^+^ cells in the lungs and pulmonary lymph nodes (pLN) of Mtb-infected C57BL/6 mice. However, they were unable to detect production of the characteristic regulatory cytokine IL-10 by this cell population *in vitro*. These same researchers (51) showed that Tregs expand both in lungs and pLN; but this occurs after Mtb has been transported to pLN, same as effector T cells. The adoptive transfer of Mtb-specific Tregs (CD4^+^CD25^+^) before T cell expansion results in higher BL, but no effects were seen when using non-specific Tregs or specific effector cells. In a later study, the authors found that the Mtb-specific Treg expansion is higher in pLN, but these cells have a short-life, and do not accumulate as infection progresses (52). These FoxP3^+^ cells form pLN have a high expression of the transcription factor Helios and expand from cells already expressing FoxP3^+^, suggesting they are thymically derived. The reduction in the Treg levels would be mediated by the transcription factor T-bet in an IL-12 dependent manner.

Contrary to these observations, a reduction in the frequency of Tregs in lungs after infection was described by McBride et al. (53) in both toll-like receptor 2 (TLR2) knockout mice (C57BL/6 strain) and their wild-type counterparts, although less in the former, which had a higher BL. In this study, the authors demonstrated that TLR2 activation on myeloid cells is necessary to induce the accumulation of CD4^+^FoxP3^+^ in the lungs, and that these accumulated Tregs prevent immunopathology and associated tissue damage. This suggested that Tregs may function by restraining the influx of monocytes and neutrophils, serving to limit the availability of niches for Mtb to replicate. In this study authors were not able to discern whether the increased BL was caused by the absence of TLR2-mediated antimicrobial activity in the granuloma or by enhanced inflammation. The latter hypothesis would support the findings in the NHP model (44) and the idea that Tregs and T effector cells act together to control inflammation without increasing Mtb replication (45). In another study, the lungs of Mtb-infected resistant mouse strains (C57BL/6 and BALB/c), compared to the DBA/2 susceptible strain, had higher numbers of Tregs as well as of CD103^+^ dendritic cells, which showed an anti-inflammatory profile (54).

The concern that Tregs might interfere with the protective effect of BCG vaccination has been addressed. C57BL/6 mice were administered an adenoviral vector that expresses murine IL-28B, which is thought to down-regulate Tregs. When this was given together with BCG and a subunit vaccine booster, Tregs were depleted in the spleen but this had no influence on the effects of vaccination (55). These results support what was previously observed by Quinn et al. (56), who inactivated Tregs with anti-CD25 mAb prior to vaccination and found that this did not affect BCG effectiveness. Nonetheless, another study of BCG booster vaccine candidates in Mtb-infected BALB/c mice showed an association between lower BL and a higher ratio of CD4^+^ to CD4^+^FoxP3^+^ in the lungs (57).

### Tregs in Human TB

The importance of the phenotypic markers used to define Tregs, discussed at the beginning of this review, is highlighted in the work carried out by Zewdie et al. (58), who found that while CD4^+^CD25^+/hi^ T-cell frequencies were higher in patients with active TB than individuals with LTBI, there were no differences in the frequency of CD4^+^CD25^+^CD127^lo^, CD4^+^CD25^+^FoxP3^+^, or CD4^+^CD25^+^FoxP3^+^CD127^lo^ T cells. A similar finding had been noted before by Wergeland et al. (59), who found lower levels of CD4^+^CD25^+^CD127^−^ Tregs, but not of CD4^+^CD25^+^FoxP3^+^ Tregs, in healthy controls compared to subjects with either active TB or LTBI. The specific parent population in the hierarchy of the analysis is also relevant. For example, in one study the percentage of CD4^+^CD25^hi^ cells expressing FoxP3 was the same in both study groups, but the percentage of CD4^+^CD25^hi^FoxP3^+^ relative to total CD4^+^ cells was significantly different between healthy donors and patients with pulmonary TB (PTB) (60).

The first association between Tregs and TB patients was shown by Guyot-Revol et al. (61). Before starting treatment, TB patients had higher frequencies of CD4^+^CD25^hi^ Tregs and a higher expression of FoxP3 in PBMCs compared to healthy controls. This difference has been shown in various cohorts summarized below.

Burl et al. (62) performed a study of FoxP3 gene expression in TB contacts. They found that non-infected contacts (ELISPOT- and tuberculin skin test (TST)-negative) had higher levels of FoxP3 mRNA in blood than Mtb-infected contacts (ELISPOT- and TST-positive), but lower levels than patients with active TB. They hypothesize that this may be due to the sequestering of Tregs in the lungs at early stages of infection and their subsequent reappearance in the periphery. However, it has been shown that in patients with PTB, but not in LTBI subjects, Treg levels are higher in bronchoalveolar lavage (BAL) than in blood (63). FoxP3 expression was detected in the immunohistochemical analysis of Mtb-infected lymph nodes (LN) (60). In patients with LN TB, there were higher levels of Tregs and activated CD4^+^ T cells among lymph node mononuclear cells than among PBMCs (64). Higher levels of Tregs have also been described in pleural effusion fluid compared to PBMCs (65, 66).

Chiacchio et al. found no differences in Treg levels unless they did an Mtb-specific *in vitro* stimulation. After 1- or 6-day stimulation with Mtb-specific RD1 proteins, TB patients had higher levels of both CD4^+^CD25^hi^FoxP3^+^ and CD4^+^CD25^hi^CD39^+^ Tregs than healthy controls (29). In PBMCs stimulated with the mycobacterial antigens PPD, ESAT-6, or CFP-10, the characterization of activated CD4^+^ T cells showed a higher percentage of CD4^+^CD25^+^CD134^+^-expressing CD39 in active TB than in LTBI. In line with the high expression of this immunosuppressive molecule, the ratio of IL-10 to IFN-γ production was increased in cell cultures from active TB cases compared to LTBI (67). The characterization of BCG-stimulated whole blood in a pediatric TB population supports these results: Tregs were higher in TB than in healthy controls (68). Moreover, the analysis of cytokines in the supernatants of these cultures showed higher levels of pro-inflammatory mediators in healthy controls. When comparing PTB to extrapulmonary TB, the authors found that Tregs could not be used to distinguish the two populations. Interestingly, however, when they looked at Treg levels after 6 months of treatment, they found that children with PTB had a significant increase but those with extrapulmonary TB did not.

Xu et al. (69) studied PTB patients at the time of diagnosis (sputum smear positive) and the same subjects after 3 weeks of treatment (sputum smear negative). Like Whittaker et al. (68), they found that with the course of treatment, both Treg frequency in PBMCs and plasma levels of IL-10 increased, and at the same time correlated negatively with plasma levels of IL-17. In contrast, Singh et al. (70) described a positive correlation between Tregs and BL in sputum.

A follow-up study performed over 9 months, comprising the 6 months of treatment, showed that Tregs continued to increase until month 4, but later decreased until levels matched those of healthy controls (71). Two similar studies with 2- or 6-month follow-up periods during treatment showed that Treg levels were higher in pulmonary TB patients than healthy controls, and decreased with chemotherapy, with no transient increase (72, 73). Agrawal et al. (74) suggest that this could be a way to monitor treatment success or failure, since the one case of failure they had in a new cohort had had an increase in Treg levels. In fact, the lack of Treg decrease with treatment has been linked to multidrug-resistant TB (MDR-TB) and has been proposed for monitoring purposes (70). Nevertheless, the PBMCs stimulus with BCG antigens showed higher levels of Tregs in TB patients but also in cured individuals when comparing with LTBI (75). Prophylactic treatment of LTBI subjects resulted in augmented levels of Tregs with the phenotype CD4^+^CD25^+^FoxP3^+^, but no differences were seen in levels of CD4^+^CD25^+^CD127^−^ (59). However, the latter phenotype was the one that differentiated between control subjects and those with TB or LTBI.

Regarding the importance of the balance between pro- and anti-inflammatory responses, Luo et al. (76) found that not only were Treg percentages higher in patients with active TB than in those with LTBI or controls, but so were Th17 cell percentages (and their corresponding cytokines in plasma). Furthermore, LTBI patients presented higher levels of Tregs and IL-10 than healthy controls. Conversely, Chen et al. (65) found that the blood of TB patients had higher levels of FoxP3^+^ CD4^+^ cells but lower levels of IL-17-producing CD4^+^ cells compared to that of both healthy donors and LTBI subjects. Nevertheless, IL-6 and TGF-β plasma levels were higher, a fact typically linked to induction of the Th17 population. The authors suggest a possible explanation in terms of reduced expression of IL-6R CD4^+^ cells caused by Mtb.

Pang et al. (77) also found a higher frequency of Tregs in blood in active TB than in LTBI or healthy controls. When they took a closer look at this group of subjects, they observed that Treg levels were higher in patients with a positive sputum smear and in those with cavitary PTB. A different approach was taken by Lim et al. (78), who looked into the differences between drug-sensitive TB and MDR-TB. They did not find significant differences between these groups, but once again Treg levels were higher in active TB than in healthy controls. Treg frequency was higher in smear-positive patients and did not correlate with radiological extent or cavitation. MDR-TB patients whose sputum results had remained negative for at least 6 months had Treg levels similar to those of healthy controls. More recently, research with the same target population showed that MDR-TB patients had higher Treg frequencies than those with drug-sensitive TB, and this was supported by blood levels of regulatory cytokines IL-10, and TGF-β (79). The same differences were found when looking at CD4^+^CD25^+^ cells expressing the co-stimulatory receptors PD-1 or CTLA-4. Similarly, patients with MDR-TB had higher (though not significantly higher) percentages of CD4^+^CD25^+^ Tregs in blood and higher IL-10 levels in serum than those with drug-sensitive TB (80). We note that neither of these studies provided any data on the extent of pulmonary disease, which usually is worse in MDR-TB (81).

## Concluding Remarks

It is clear that the balance between pro- and anti-inflammatory responses to Mtb infection is important in the development of the disease and should be further studied.

Research performed on animal models is a useful way of focusing on different aspects in the progression of Mtb infection and their relationship with Tregs. One of the main advantages of these studies is the possibility of easily looking into the site of infection and lymphoid tissues. They also provide the possibility of modulating many of the involved actors to further understand the mechanisms behind Mtb infection. In this sense, the development of knock outs and monoclonal antibodies has been of great help. However, using animal models has obvious limitations when the time comes to translate findings to human TB. It is crucial to set appropriate objectives in each model and to contextualize results.

Most work carried out on animal models describes an increase in Treg levels as Mtb infection progresses. Experimental depletion of CD25+ T cells as a proxy of Treg deletion, even being a rough approach to the issue, resulted in worse pathology or higher bacillary load, arguing they have in fact a detrimental role for the host. Nonetheless, higher frequencies of Tregs have been related to less susceptible mouse strains and better lung pathology or non-progression toward disease in NHP.

There have been many studies carried out in patient cohorts, with the main limitation being that they are mostly based on PBMCs. It has been shown that in TB patients Treg levels are higher at the site of infection, so the study of PBMCs may seem somewhat inaccurate. Furthermore, even when contact trace studies may give an approximate time of exposure to Mtb, the exact steps of infection are hard to elucidate in human TB. Longitudinal studies, the ones that could really shed more light on whether Tregs have a beneficial or detrimental role in TB, are rare. Potentially, monitoring of Tregs as an exploratory endpoint in future prospective cohort study addressing progression from LTBI toward active TB could be of interest to solve this question.

Many studies suggest the potential use of Tregs as a diagnostic method for active TB. In general, Tregs are higher in active TB than in LTBI or healthy controls. However, the difference between LTBI and non-infected persons is less clear. So far this seems unrealistic and much work remains to be done in this context, not only because of the everlasting issue of Treg phenotyping, but also because it is difficult to set a cut-off value for this parameter. Tregs have also been suggested as a way of monitoring the effectiveness of treatment. This option is even more challenging as direct correlation between bacillary load and Treg has never been done in the context of chemotherapy.

**Figure 1:**
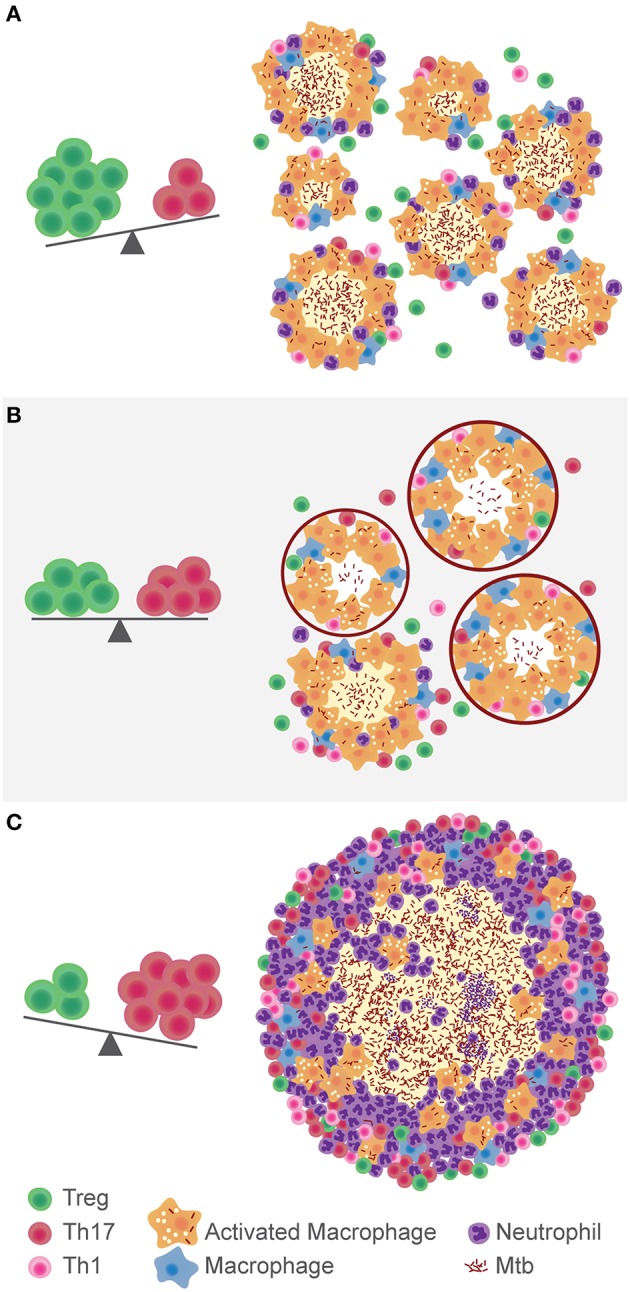
Hypothesis on the role of the balance between Tregs and Th17 cells in the development of TB. In a context of immunosuppression, where Tregs are predominant over Th17, Mtb disseminates more easily **(A)**. The immune balance between these populations gives place to the encapsulation and control of lung lesions **(B)**. If there is a high Th17 response, inflammation and neutrophils recruitment fuel the growth of the granuloma and development of TB **(C)**.

All in all, the research carried out so far on Tregs and TB suggests it is not a black-and-white issue. In a setting of excessive inflammation, they would appear to be beneficial for the host. However, their immunosuppressive role could also be advantageous for the pathogen. It is most probably a matter of balance between both responses (Figure 1). Mtb is a slow growing pathogen that generates a delayed immune response. The scale tipped toward Tregs at this instance would play in favor of the bacillus, allowing its dissemination, and intracellular growth. Also, the relatively low Th17 response would delay the recruitment of neutrophils to the site of infection. However, in the opposite scenario, a low Treg response would benefit the onset of an excessive inflammatory response, characteristic of pulmonary TB. Ideally, a balanced situation in which the immune system controls Mtb growth without generating tissue damage, would allow the encapsulation of the small granulomas and limit Mtb spread. More efforts should be done on analyzing not just the regulatory response against Mtb, but rather its equilibrium with the pro-inflammatory response, especially Th17.

## Author Contributions

PC and P-JC conceptualized the review. PC provided an initial draft. P-JC performed the final edits.

### Conflict of Interest Statement

The authors declare that the research was conducted in the absence of any commercial or financial relationships that could be construed as a potential conflict of interest. P-JC is co-founder of two spin-offs related to tuberculosis: Archivel Farma and Manremyc.
